# Subject-specific one-dimensional fluid dynamics model of chronic thromboembolic pulmonary hypertension

**DOI:** 10.21203/rs.3.rs-3214385/v1

**Published:** 2023-08-03

**Authors:** Amirreza Kachabi, Mitchel J. Colebank, Naomi Chesler

**Affiliations:** University of California, Irvine; University of California, Irvine; University of California, Irvine

**Keywords:** Pulmonary hypertension, Hemodynamics, Computational fluid dynamics, Image-based modeling, Wall shear stress

## Abstract

Chronic thromboembolic pulmonary hypertension (CTEPH) develops due to the accumulation of blood clots in the lung vasculature that obstruct flow and increase pressure. The mechanobiological factors that drive progression of CTEPH are not understood, in part because mechanical and hemodynamic changes in the pulmonary vasculature due to CTEPH are not easily measurable. Using previously published hemodynamic measurements and imaging from a large animal model of CTEPH, we developed a subject-specific one-dimensional (1D) computational fluid dynamic (CFD) models to investigate the impact of CTEPH on pulmonary artery stiffening, time averaged wall shear stress (TAWSS), and oscillatory shear index (OSI). Our results demonstrate that CTEPH increases pulmonary artery wall stiffness and decreases TAWSS in extralobar (main, right and left pulmonary arteries) and intralobar vessels. Moreover, CTEPH increases the percentage of the intralobar arterial network with both low TAWSS and high OSI. This subject-specific experimental-computational framework shows potential as a predictor of the impact of CTEPH on pulmonary arterial hemodynamics and pulmonary vascular mechanics. By leveraging advanced modeling techniques and calibrated model parameters, we predict spatial distributions of flow and pressure, from which we can compute potential physiomarkers of disease progression, including the combination of low mean wall shear stress with high oscillation. Ultimately, this approach can lead to more spatially targeted interventions that address the needs of individual CTEPH patients.

## Introduction

Chronic thromboembolic pulmonary hypertension (CTEPH) occurs when blood clots lodge in the small blood vessels of the lung and fail to fully dissolve or resolve over time. The resulting blood flow obstruction leads to an elevation in blood pressure within the pulmonary circulation. If left untreated, CTEPH causes right ventricular failure and death. With early diagnosis, however, CTEPH can be successfully treated with surgery (Feinstein et al. 2001). CTEPH is diagnosed by invasive right heart catheterization (RHC) to measure mean pulmonary artery pressure (mPAP) ≥ 20 mmHg and pulmonary capillary wedge pressure (PCWP) ≤ 15 mmHg, with subsequent imaging to confirm clot burden(Lang et al. 2014).

A key knowledge gap in CTEPH is the drivers of clot growth and dissolution over time (Tsubata et al. 2023). Vascular mechanobiological factors are thought to play an important role. Abnormalities in the magnitude and directions of wall shear stress (WSS) contribute to pathological processes such as endothelial dysfunction, inflammation, and impaired vasodilation (Allen et al. 2023)WSS in small pulmonary arteries likely impact clot organization and growth as well as dissolution (Tsubata et al. 2023). Given that pulmonary artery stiffness increases with pulmonary hypertension development (Wang and Chesler 2011) and alters flow and thus shear stress dynamics (Su et al. 2013), pulmonary artery stiffness may also contribute to CTEPH progression. Therefore, computing WSS and vascular mechanics throughout the pulmonary arterial network can provide useful information regarding likely sites for disease progression.

Large animal models of CTEPH have advanced our understanding of the pathophysiology of CTEPH (Mulchrone et al. 2019; Stam et al. 2021), but typically do not report local mechanical stimuli for disease progression such as WSS. Image-based computational fluid dynamics (CFD) modeling can compute pulmonary artery blood flow with high spatial resolution (Burrowes et al. 2017; Bordones et al. 2018; Wang et al. 2020), from which WSS can be calculated. The combination of experiments in large animal models of CTEPH with CFD analysis enables estimation of mechanobiological stimuli in sections of the pulmonary arterial network inaccessible to measurement. Moreover, this combination of techniques can be used to compute stimuli that cannot be measured from imaging alone, such as pressure distribution.

Here, we use an image-based nonlinear one-dimensional (1D) CFD model to predict hemodynamics throughout the pulmonary arterial network before and after CTEPH. The large animal data were previously published by (Mulchrone et al. 2019). We calibrate our CFD model to the hemodynamic and imaging data available and then use the results to quantify the impact of CTEPH on metrics of WSS including time-averaged WSS (TAWSS) and oscillatory shear index (OSI). We also quantify the impact of CTEPH on wall stiffness. This synergistic experimental-computational framework gives insight into the extralobar and intralobar mechanobiological effects of CTEPH with subject specificity and provides a future tool for understanding the local determinants of disease progression seen clinically.

## Methods

### Animal Study

All experimental data were obtained retrospectively; a detailed protocol and complete reporting of measurements can be found in (Mulchrone et al. 2019). Briefly, CTEPH was induced by injecting multiple microspheres into the pulmonary arteries (PAs) using an indwelling catheter following methods established by Hori et al.(Hori et al. 2012) in five adult male canines (12 ± 1 kg body weight). Magnetic resonance imaging (MRI) and RHC were performed under anesthesia sustained by administering 1–3% isoflurane in 100% oxygen, and ventilation regulated to maintain end-tidal CO_2_ levels within the range of 30–50 mmHg. Instrumentation details are available in (Bellofiore et al. 2013).

### Hemodynamic and Imaging data

All measured hemodynamic data were collected at baseline and after animals reached an mPAP $\left({\overset{‾}{P}}_{PA}\right)\geq 25$ (based on clinical guidelines when these experiments were conducted (Lang and Madani 2014)) and mean PCWP $\left({\overset{‾}{P}}_{PCW}\right)\leq 15\;mmHg$ as detailed in (Bellofiore et al. 2013). Pressure data included systolic and diastolic main pulmonary artery (MPA) pressures and PCWP. Imaging data including time-series velocity and area in the MPA, left (LPA), and right (RPA) pulmonary arteries (i.e., the extralobar PAs) were obtained from MRI. Time-series flow, , $Q\left(t\right)$ (mL/s), was calculated as the product of velocity and cross-sectional area for each extralobar PA.

From this set of measurements, the following were used as input data : $\left\{{\overset{‾}{P}}_{PA},{\overset{‾}{P}}_{PCW},Q_{MPA}(t)\right\}$ and the following were used as calibration data: $\left\{P_{sys},P_{dia},A_{sys},A_{dia},Q_{LPA}(t),Q_{RPA}(t)\right\}$, where $P_{sys},\;A_{sys},\;P_{dia}$, and $A_{dia}$ are the pressures and areas in the MPA at systole and diastole, respectively, ${\overset{‾}{P}}_{PA}=1/3P_{sys}+2/3P_{dias}$ is the mean MPA pressure, $Q_{MPA}(t)$, $Q_{LPA}(t)$ and $Q_{RPA}(t)$ are the MPA, LPA, and RPA flows, which are functions of time. The time-averaged MPA flow, ${\overset{‾}{Q}}_{MPA}$ is also used with ${\overset{‾}{P}}_{PA}$ and ${\overset{‾}{P}}_{PCW}$ to compute overall pulmonary vascular resistance as $R=\left({\overset{‾}{P}}_{PA}-{\overset{‾}{P}}_{PCW}\right)/{\overset{‾}{Q}}_{MPA}$.

### CFD Model Geometry

**Figure 1:**
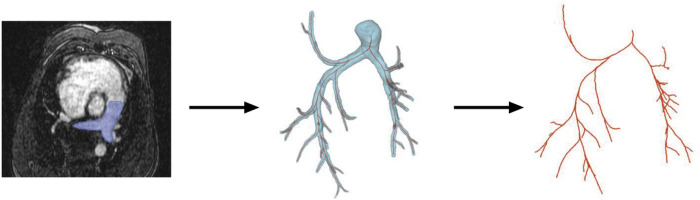
Process for extracting 1D arterial network for one subject, including (a) arterial identification from MRI images, (b) 3D segmentations of the arterial network and (c) network extraction obtained by conversion from the 3D segmentations.

We created a 1D arterial network model as the computational domain for each subject pre- and post-CTEPH, shown for a representative subject in Fig. 1. First, from sagittal plane MR images (Fig. 1a), 3D segmentations of extralobar and intralobar PA geometries were created using 3D slicer (Fedorov et al. 2012) (Fig. 1b). We then used the vascular Modeling Toolkit to convert the 3D segmentations to 1D centerline networks (Fig. 1c) (Antiga et al. 2003). Finally, we post-processed the specific networks using custom MATLAB (Natick, MA) software to create the 1D CFD mathematical domain for simulations (Colebank et al. 2021). To ensure agreement between the dynamic extralobar PA area in the model and the time-averaged extralobar PA area from MRI, we scaled the extralobar PA area in the network geometry at diastole to match the MRI data at diastole.

### Governing Equations

We use time-dependent, 1D fluid dynamics equations to simulate hemodynamics in the pulmonary arterial networks. We assume all blood vessels are straight, cylindrical, and impermeable. Blood flow through the arterial network is assumed to be axisymmetric, incompressible, Newtonian, and laminar such that the governing equations are:

1 $$\frac{\partial Q}{\partial x}+\frac{\partial A}{\partial t}=0$$

2 $$\frac{\partial Q}{\partial t}\;+\;\frac{\left(\gamma \;+\;2\right)}{\left(\gamma +1\right)}\frac{\partial }{\partial x}\left(\frac{Q^{2}}{A}\right)\;+\;\frac{A}{\rho }\frac{\partial P}{\partial x}=-\frac{2\pi \mu (\gamma \;+\;2)}{\rho }\frac{Q}{A}$$

where x(cm) and t(s) represent the axial and temporal coordinates, and P(x,t) is the transmural blood pressure (mmHg)(Olufsen et al. 2000). Q(x,t) is the volumetric flow (mL/s) and $A(x,t)=\pi (r(x,t){)}^{2}$ is cross sectional area (cm^2^) where r(x,t) is the spatially and temporally-dependent radius of the artery (cm). The density (ρ) and dynamic viscosity (μ) of blood are assumed to be constant and equal to 1.03 (g/mL) and 0.03 (Poise), respectively (Kamiya and Togawa 1980). Further we assume the fluid velocity at the wall is equal to the velocity of the wall (no-slip condition).

The 1D theory requires an explicit expression for the fluid velocity profile. Here, we use the power-law profile:

3 $$u(x,t)=\overset{‾}{U}(t)\frac{\gamma \;+\;2}{\gamma }\left(1-\frac{r^{\gamma }}{r(x,\;t{)}^{\gamma }}\right)$$

where u(x,t) is the blood velocity (cm/s), $\overset{‾}{U}(t)$ is the mean velocity (cm/s), and γ is the power-law constant, which is set to 9 to provide a nearly uniform velocity profile within the center of the vessel consistent with a high Womersley number (Alastruey et al. 2008; Spilker and Taylor 2010).

### Wall Mechanics

Within the given 1D equations, there are three dependent variables, {P(x,t),A(x,t),Q(x,t)}. To close the system of equations, a constitutive model for the artery wall is required. We used a linear elastic model (Safaei et al. 2016), which assumes a linearly elastic, thin walled, isotropic, and incompressible material in the form of a cylinder. Under these circumstances, the constitutive law can be written as:

4 $$P(x,t)=\frac{4}{3}\frac{Eh}{r_{0}}\left(\sqrt{\frac{A(x,\;t)}{A_{dia}}}-1\right)+P_{dia}$$

where E is the circumferential Young’s modulus (mmHg), h (cm) is the wall thickness, and $A_{dia}=\pi r_{dia}^{2}$ (cm^2^)is the lumen area at the diastolic pressure $P_{dia}$ (mmHg). The term $\frac{Eh}{r_{0}}$ accounts for both structural and load-dependent stiffening and is a potential physiomarker of disease severity. We calculated this term analytically for individual subjects pre- and post-CTEPH using systolic pressure and area in the MPA in Eq. (4). Finally, model equations were solved using two-step Lax-Wendroff method, detailed in prior work (Olufsen et al. 2000).

### Boundary Conditions

Boundary conditions are specified at each vessel inlet and outlet. We used the $Q_{MPA}(t)$ as the inlet flow boundary condition in the MPA. At extralobar and intralobar vessel junctions, flow conservation $Q_{p}=Q_{d1}+Q_{d2}$ and pressure continuity $P_{p}=P_{d1}=P_{d2}$ were enforced, where the subscript ‘ p ‘ denotes the parent vessel that bifurcates into two daughter vessels ‘d1’ and ‘d2’.

At each terminal intralobar vessel, we used 3 element Windkessel models to represent all vessels outside those obtained by segmentation. Each Windkessel model includes a proximal resistance $\left(R_{p}\right)$, distal resistance $\left(R_{d}\right)$, and compliance (C). Nominal Windkessel resistances are calculated by distributing the total pulmonary vascular resistance, $\left(P_{mean}-P_{PCW}\right)/{\overset{‾}{Q}}_{MPA}$, using a Poiseuille argument (Qureshi et al. 2019), i.e. $R_{T}=8\mu L/\pi r^{4}$. We assume that the proximal and distal resistances are initially equivalent, i.e. $R_{p}=R_{d}=R_{T}/2$. The determination of C requires fitting an exponential decay function to the change in $P_{MPA}$ from systole to diastole (Qureshi et al. 2019). Total proximal and distal resistance in the network is calculated from the sum of the resistances in parallel, while the total compliance in the network is calculated as using circuit theory of capacitators in parallel (Qureshi et al. 2019).

In the experimental data set used here, Mulchrone et al.(2019) previously observed an asymmetric distribution of obstruction with the majority of blockage in the left lung. Because less contrast then flowed into the left lung, imaging resolution was poorer on this side. To account for signal loss, the flow into the left lung in post-CTEPH models was recalculated as ${\tilde{Q}}_{LPA}(t)=Q_{MPA}(t)-Q_{RPA}(t)$ and then used to calculate the left lung Windkessel resistance and compliance distributions.

### Model Calibration

**Figure 2:**
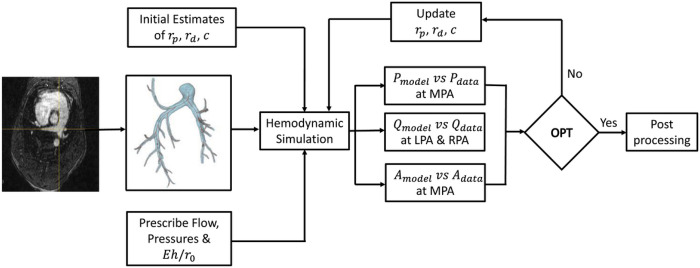
Schematic of model calibration process for Windkessel parameters with experimental pressure, area and flow data.

Windkessel parameters were inferred based on the set of calibration data $\left\{P_{sys},P_{dia},A_{sys},A_{dia},Q_{LPA}(t),Q_{RPA}(t)\right\}$ at baseline and $\left\{P_{sys},P_{dia},A_{sys},A_{dia},{\tilde{Q}}_{LPA}(t),Q_{RPA}(t)\right\}$ post-CTEPH. Given the limited data and numerous Windkessel elements (i.e., $R_{p},\;R_{d}$ and C for each terminal branch), the Windkessel parameters were scaled by the same set of scaling factors $\backslash varvec\theta_{wk}=\left\{r_{p},r_{d},c\right\}$ using weighted, nonlinear least squares (Qureshi et al. 2019). We defined a cost function in which the model predictions are matched to the calibration data (and denoted by a superscript ) by minimizing:

5 $$S(\backslash \mathrm{varvec}\theta )=\sum_{j}{\left(P_{j}^{c}-P_{j}\left(\backslash \mathrm{varvec}\theta_{wk}\right)\right)}^{2}+\sum_{j}{\left(A_{j}^{c}-A_{j}\left(\backslash \mathrm{varvec}\theta_{wk}\right)\right)}^{2}+\frac{1}{N}\sum_{k}\sum_{i=1}^{N}{\left(Q_{k}^{c}\left(t_{i}\right)-Q_{k}\left(t_{i};\backslash \mathrm{varvec}\theta_{wk}\right)\right)}^{2}$$

where $P_{j}^{c}$ is the calibration pressure for $j=sys,dia,P_{j}\left(\backslash varvec\theta_{wk}\right)$ is computed pressure, $A_{j}^{c}$ is the calibration area, and $A_{j}\left(\backslash varvec\theta_{wk}\right)$ is the computed area. For the time-series data terms, N is the length of the time vector spanning one cardiac cycle, $Q_{k}^{c}\left(t_{i}\right)$ denotes calibration flow data for k=LPA,RPA, and $Q_{k}\left(t_{i};\theta_{wk}\right)$ is the computed time-series flow at time $t_{i}$. We used the function Isqnonlin in MATLAB to identify the global minimum for our objective function based on ten randomized starting values (Qureshi et al. 2019). The calibration process is shown in Fig. 2.

### Wall shear stress

The TAWSS and OSI were computed as

$$TAWSS=\frac{1}{T}\int_{0}^{T}|\tau (x,t)|dt$$

where

(6) $$\tau (x,t)=\mu {\left(\frac{\partial u(x,t)}{\partial r(x,t)}\right)}_{r=R}$$

and

7 $$OSI=0.5\left(1-\frac{\frac{1}{T}\left|\int_{0}^{T}\tau (x,\;t)dt\right|}{\frac{1}{T}\int_{0}^{T}|\tau (x,\;t)|dt}\right)$$

throughout the arterial network. Here, τ(x,t) (dyne/cm^2^) is the spatially and temporally dependent arterial WSS and T(s) is the cardiac cycle length. Note that whereas TAWSS and OSI in the large, extralobar arteries are straightforward to compare from baseline to CTEPH, the number and size of intrapulmonary arteries detectable in the imaging, and thus represented in the 1D model, varies from baseline and CTEPH. To enable comparison from baseline to CTEPH, we report TAWSS and OSI averaged over all intralobar arteries for each subject.

### Statistical Test

Paired one-sided t-tests (in Excel) were used to assess significance with p<0.05.

## Results

The objective of this study was to formulate a comprehensive framework that leverages clinically measurable hemodynamic data and imaging to forecast non-clinically measurable hemodynamic and mechanical parameters relevant to pulmonary vascular disease progression. We used imaging data to build subject-specific geometries of the pulmonary arterial network for fluid dynamics simulation pre- and post-CTEPH induction. After calibrating to hemodynamics pre- and post-CTEPH, we performed 1D fluid dynamics model simulations to investigate the impact of CTEPH on spatial and temporal distributions of wall shear stress and vascular mechanics.

### Model calibration

**Figure 3:**
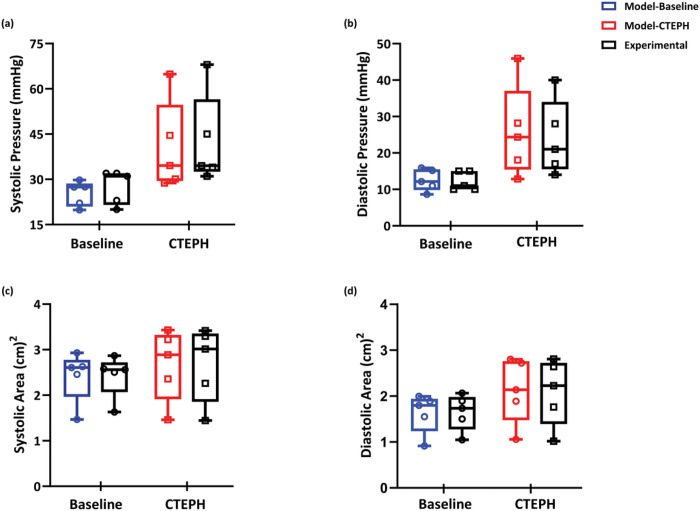
Comparison between measured data and model prediction. (a) systolic pressure, (b) diastolic pressure, (c) Systolic are and (d) diastolic area.

Model fits to scalar calibration data $\left\{P_{sys},P_{dia},A_{sys},A_{dia}\right\}$ at baseline and CTEPH are shown in Fig. 3. Following the development of CTEPH, all subjects had increased systolic and diastolic pressures as expected (Fig. 3a and b). Four out of five subjects had increased systolic and diastolic areas. We attribute the one exception to low image resolution at baseline, which led to a non-significant group increase with CTEPH (Fig. 3c and d).

**Figure 4:**
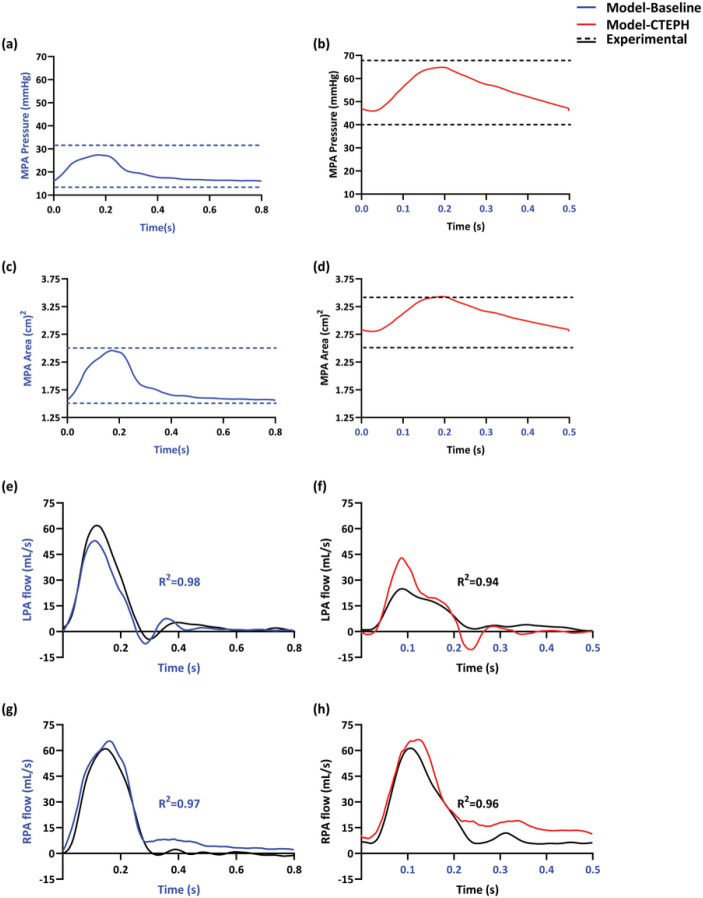
Measured hemodynamics and model predictions for a representative subject. Figure 3(a,b) shows model pressure predictions (solid lines) compared to measured systolic and diastolic pressure (dashed lines) for a representative animal in which mPAP increased from 20 to 51 mmHg from baseline (blue; left) to CTEPH (red; right). Figure 3(c,d) illustrates the predicted areas values from the model (solid lines) and the measured systolic and diastolic pressures at MPA (dashed lines) for the same animal. The animal exhibited an increase in mean area from 1.75 to 3.2 (cm^2^) from the baseline (blue; left) to the CTEPH (red; right) condition. For the same animal, Figures 2(e,f) and 2(g,h) show LPA flow and RPA flow, respectively, from measurements (black) and model predictions (blue, left at baseline and red, right with CTEPH). In this animal, a notable decrease in mean flow at LPA was observed after CTEPH induction, decreasing from 10.02 to 7.40 (mL/s), which represents a reduction of approximately 26%. Conversely, an increase in mean flow of approximately 46% was observed in the right lung.

Agreement between model simulations and the time-series experimental measurements $\left\{Q_{LPA}(t),Q_{RPA}(t)\right\}$ were assessed by their R^2^ value, which were 0.94 ± 0.05 and 0.98 ± 0.01 in the LPA and RPA, respectively, at baseline. The R^2^ values were 0.93 ± 0.03 and 0.96 ± for the LPA and RPA, respectively, after CTEPH. As previously observed for this experimental data set (Mulchrone et al. 2019), all subjects had a reduced cardiac output and significant asymmetries in flow distribution after CTEPH. Representative model results and comparison to experimental data are shown for a single subject at baseline and after CTEPH (Fig. 4).

### Pulmonary Arterial Network Properties

**Figure 5:**
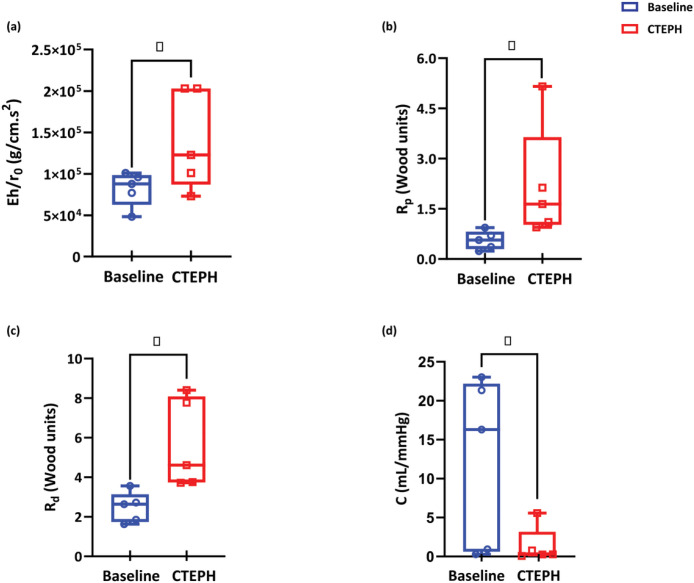
Changes in the material stiffness terms (a), proximal resistance (b), distal resistance (c) and compliance (d) from Base (blue) to CTEPH(red)

Pulmonary arterial stiffness was analytically computed for each subject using $\left\{P_{sys},P_{dia},A_{sys},A_{dia}\right\}$ in Eq. (4). As shown in Fig. 5a, CTEPH significantly increased $\frac{Eh}{r_{0}}(p=0.02)$, which can be attributed to the combination of structural and load-dependent stiffening. Similarly, CTEPH significantly increased Windkessel total proximal resistance $R_{p}(p=0.049)$ and total distal resistance $R_{d}(p=0.035)$ (Fig. 5b and c) and significantly decreased Windkessel total vascular compliance C(p=0.033) (Fig. 5d).

### Wall Shear Stress

**Figure 6:**
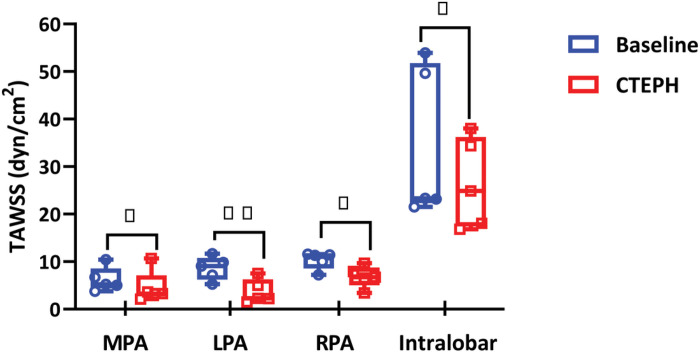
MPA, LPA, RPA and Intralobar arteries TAWSS under baseline (blue) and CTEPH (red) conditions; n=5

With the model predictions of {P(x,t),A(x,t),Q(x,t)}, changes in TAWSS with CTEPH were computed (Fig. 6). In four out of five subjects, TAWSS decreased at the MPA and RPA; in all subjects, TAWSS decreased in the LPA and most intralobar arteries. One subject exhibited an increase in MPA TAWSS (by ≈3%) and one subject exhibited an increase in RPA TAWSS (by ≈9%) following CTEPH. On average, there was a significant decrease in TAWSS in the MPA, LPA, RPA, and intralobar vessels (p=0.02,p=0.007,p=0.04 and p=0.02, respectively).

**Figure 7:**
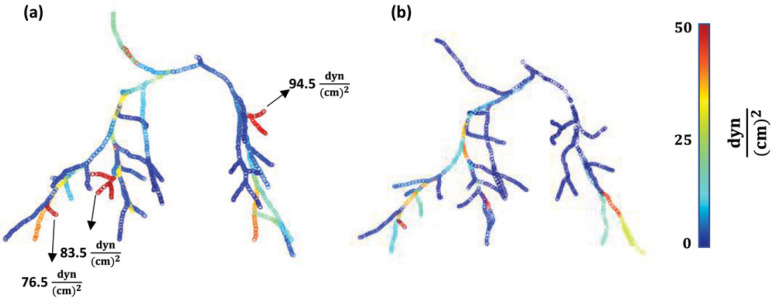
3D map of TAWSS distribution across the entire arterial network for a representative animal at baseline (a) and CTEPH (b). This map demonstrates the changes in TAWSS that occurred in response to CTEPH induction throughout different segments of the network.

A three-dimensional map displaying the distribution of TAWSS across the entire arterial network for a representative subject at baseline and CTEPH is shown in Fig. 7. At baseline, TAWSS values ranged from 1.7 to 94.5 (dyne/cm^2^) with a mean value of 22.6 whereas after CTEPH, TAWSS decreased, ranging from 1.4 to 64.3 (dyne/cm^2^) with a mean value of 16.1.

**Figure 8:**
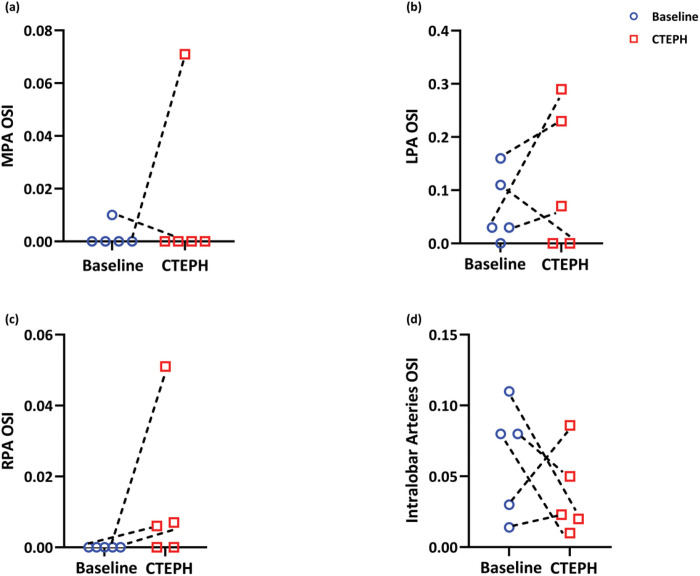
MPA (a), LPA(b), RPA (c) and Intralobar arteries (d) OSI under baseline (blue) and CTEPH (red) conditions; n=5.

Model flow simulations also enabled computation of OSI distribution. With CTEPH, OSI increased and decreased in the extralobar (MPA, LPA, and RPA) and intralobar arteries without a consistent pattern (Fig. 8). For several subjects, MPA and RPA OSI values were essentially zero.

**Figure 9:**
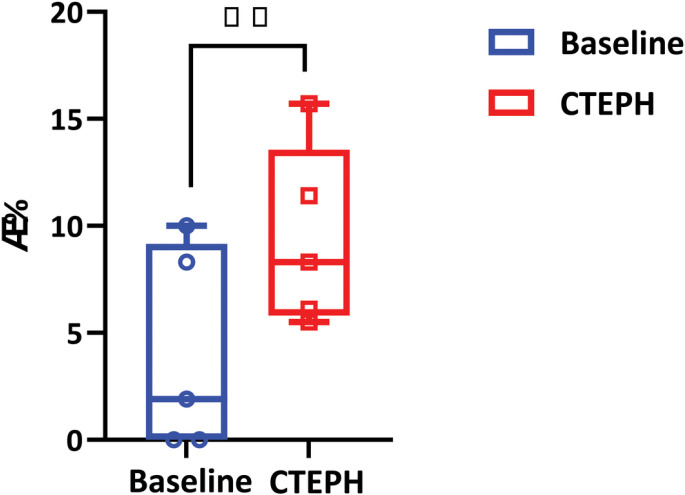
The percentage of vessels that have TAWSS<5 dyn/cm and OSI>0.05 at baseline (blue) and CTEPH (red) conditions; n=5.

To better understand the hemodynamic impact of CTEPH, we investigated changes in a combined metric using the spatial distribution of TAWSS and OSI. We identified regions of the network with both low TAWSS (< 5 dyne/cm^2^) and high OSI (> 0.05); the TAWSS cutoff was based on previous literature (Li et al. 2009) and the OSI cutoff was based on the average OSI for all vessels across all subjects. As shown in Fig. 9, the percentage of regions that met this criterion, *ϕ*, significantly increased with CTEPH (p=0.0004).

## Discussion

In this study, we used an image-based nonlinear one-dimensional CFD model to predict arterial hemodynamic features that would be difficult, if not impossible, to measure experimentally and which may be clinically relevant to disease progression. Based on an existing set of imaging data pre- and post-CTEPH, we developed subject-specific 1D arterial network models. We used an efficient multi target optimization scheme for calibration that incorporated experimentally measured MPA systolic and diastolic pressures and areas, as well as dynamic flow data at the LPA and RPA. Our approach yielded simulation results with excellent agreement with measured data, and predicted pulmonary arterial stiffness, TAWSS and OSI distribution pre- and post-CTEPH. The increase in pulmonary arterial stiffness post-CTEPH will drive upstream (i.e., right ventricular (Liu et al. 2022)) and downstream (i.e., pulmonary capillary (Wang and Chesler 2011)) remodeling to accelerate disease progression. Moreover, changes in WSS are relevant to the growth or dissolution of thrombi (Sakariassen et al. 2015). Thus, these model results allow for a more comprehensive understanding of the effects of CTEPH, enhancing our ability to study this condition and potentially improve patient care and treatment strategies.

### Modeling and Optimization

Hemodynamics modeling is a powerful tool for investigating hypothesized mechanisms of pulmonary vascular disease development and progression. There has been limited application of these tools to CTEPH in comparison to other forms of pulmonary hypertension, such as pulmonary arterial hypertension (PAH) (Clipp and Steele 2009; Zambrano et al. 2018; Dong et al. 2021). The use of subject-specific modeling has the potential to provide clinicians with a better understanding of patient specific disease states, optimizing clinical decision-making and ultimately enabling personalized medicine. Several computational studies calibrated their models using existing experimental data. Zambrano et al. (2018) developed subject-specific computational models of pulmonary vascular hemodynamics for both a PAH patient and a healthy individual. The parameters of the model, including Windkessel parameters ($R_{p},\;R_{d}$,and C) for each terminal vessel in the outflow branches, were adjusted using in vivo measurements of pressure from RHC. Additionally, the linearized stiffness E of the large arteries was adjusted based on the relationship between pressure and diameter from MRI. Clipp and Steele (2009) generated 1D CFD models of the pulmonary arterial networks of lambs, incorporating considerations such as geometry, compliance, structured tree boundary conditions, and respiration. Similar to Zambrano et al. (2018), parameters describing the boundary conditions in the Clipp et al. study were inferred by comparing the computed inlet pressure waveform to experimental data. In this study, we not only used pressure calibration data (systolic and diastolic) to infer boundary conditions but also used area (systolic and diastolic) and flow (time-series) calibration data, enhancing model accuracy and reliability. As shown in Fig. 4 for a representative animal and consistent with all animals, model predictions quantitatively matched measured pressures and flows (high *R*^2^ values).

As mentioned earlier, there are few studies that used computational modeling to predict hemodynamics in CTEPH. Colebank et al. (2021) developed 1D CFD models of pulmonary arterial networks based on computed tomography imaging in CTEPH patients. They simulated four different CTEPH disease cases to test physiological hypotheses related to remodeling. Tsubata et al. (2023) performed 3D CFD simulations to model subject-specific data from healthy subjects, CTEPH patients and CTEPH patients treated with balloon pulmonary angioplasty. However, neither paper had an abundance of pre- and post-CTEPH data from individual subjects. A novel aspect of our study is that we used paired data sets pre- and post-CTEPH, which enables a subject-specific interpretation of CTEPH progression.

### Pulmonary Arterial Parameters

Pulmonary hypertension is known to increase pulmonary arterial stiffness, which is measured in clinical and preclinical studies using a wide range of direct and indirect methods. Su et al(2017) used wave intensity analysis to indirectly measure pulmonary arterial stiffness in 10 healthy subjects and 21 subjects with pulmonary hypertension (11 with PAH and 10 with CTEPH). They reported that the wave reflection index in these subjects was significantly higher than in healthy controls. We analytically determined the mechanical properties of the PAs using systolic and diastolic pressure and a linear wall mechanics model. In contrast, previous studies(Qureshi et al. 2019) required parameter estimation to determine pulmonary arterial stiffness. As shown in Fig. 5a and consistent with the literature, we observed vessel stiffening after CTEPH induction, which may contribute to disease progression (Sun and Chan 2018).

Pulmonary arterial stiffening in CTEPH is likely a combination of load-dependent stiffening and structural stiffening (Wang and Chesler 2011; Pewowaruk et al. 2021). That is, the increase in pulmonary vascular resistance due to CTEPH increases pressure and thus increases the operating point of arteries on the non-linear pressure-diameter curve (Wang and Chesler 2011), called load-dependent stiffening. In addition, proliferative and fibrotic changes in the pulmonary arterial wall can cause structural remodeling that increases vessel stiffness (Kim et al.). The combination of increased resistance and stiffness elevates right ventricular afterload and contributes to the development of right heart failure(Wang and Chesler 2011). Qureshi et al. (2019) used a model similar to ours to study hemodynamic changes in normoxic and hypoxic mice. Their Windkessel resistance increased in hypoxa while Windkessel compliance decreased. Figure 5b–c illustrate an increase in Windkessel proximal and distal resistance with CTEPH, primarily attributable to the obstruction and narrowing of pulmonary arteries and arterioles, respectively. Additionally, as depicted in Fig. 5d, a decrease in Windkessel compliance occurs with CTEPH, likely due to a combination of load-dependent and structural stiffening in the distal arteries and arterioles.

### Flow and shear stress

Changes in the flow of blood within the lungs are believed to play a crucial role in the onset and worsening of pulmonary vascular diseases (Clark and Tawhai 2018). Here, changes in flow magnitude and distribution were observed in both lungs for all subjects from baseline to CTEPH. The change in distribution with CTEPH, in particular the left-right asymmetry, is due to uneven microspheres distribution (Mulchrone et al. 2019), which may not reflect the clinical disease state.

Due to these flow changes as well as artery dilation, our 1D model predicts WSS decreases throughout the arterial network with CTEPH. Clinical studies that use image-based methods to compute WSS in individuals have shown that those with PH exhibit lower WSS than normotensive subjects (Barker et al. 2015; Terada et al. 2016). Modeling studies have found the same result. Zambrano et al. (2021) performed 3D image-based, computational fluid dynamics simulations, in which interactions between blood flow and wall deformation were included and predicted that subjects with PAH have lower TAWSS than controls. Tang et al. (2011) used the same approach as Zambrano et al. (2021) and similarly predicted lower mean WSS in the proximal arteries in the PAH group. From patient-specific 3D computational fluid dynamics simulations in CTEPH, Tsubata et al. (2023) observed lower systolic WSS in CTEPH patients in comparison to controls as well as to patients after balloon pulmonary angioplasty treatment. Our results are consistent with the results of these image-based and computational studies, as shown in Fig. 6.

At the MPA, we found that TAWSS significantly decreased following CTEPH in four of the five subjects; in the one exception, that we attribute this to poor image quality at baseline that likely increased the estimated arterial network area. A significant reduction in LPA TAWSS was observed for all subjects in our study. The observed decrease in TAWSS can be attributed to a dramatic drop in flow to the left lung due to the severity of the obstruction. This finding is consistent with the suggestion that WSS reduction in the proximal PAs is an indicator of PAH disease severity (Yang et al. 2019). At the RPA, TAWSS decreased in all subjects except one (not the same subject for which MPA TAWSS increased). Overall, the decrease in TAWSS in the RPA was less dramatic and more variable than in the LPA (Fig. 6). In the intralobar arteries, TAWSS is higher compared to the extralobar arteries in both baseline and CTEPH conditions. This observation is consistent with Pillalamarri et al(2021) who found that TAWSS increased with increasing distance from the heart based on a computational investigation of patient-specific pulmonary hemodynamics across 32 subjects in distinct WHO groups of PH. The Pillalamarri et al. (Pillalamarri et al. 2021)study included one subject with CTEPH, in whom TAWSS decreased in the distal part of the network, consistent with our results.

The biological consequences of decreased WSS in the pulmonary circulation include altered endothelial cell signaling that drives vasoconstriction and the production of pro-inflammatory factors (Allen et al. 2023). Decreased TAWSS may also contribute to the synthesis and accumulation of collagen in the arterial wall and proliferation of smooth muscle cells (Ryu et al. 2022). This pulmonary arterial remodeling can result in a stiffer and less compliant pulmonary artery network (Friesen et al. 2019), which further exacerbates the severity of PH and can lead to right heart failure.

### Oscillatory shear index

Like TAWSS, OSI can drive changes in endothelial cell structure and function that affect disease progression. In endothelial cells, high OSI is associated with increased reactive oxygen species, which can drive vasoconstriction and inflammation (Peiffer et al. 2013). In the large extrapulmonary arteries, especially the MPA and RPA, OSI magnitude was quite small at baseline and only became somewhat larger with CTEPH. In contrast, Tsubata et al. (2023) found a significant increase in proximal artery OSI in CTEPH subjects compared to controls. Their OSI magnitudes were on average much larger than ours (0.05 verses 0.35). Bartolo et al. (2022) used a 1D framework similar to ours to compute OSI in a human pulmonary arteriovenous network and found OSI magnitudes similar to ours, which suggests that the 1D framework and/or the assumed velocity profile affects OSI calculations.

In the intralobar arteries, there was no clear pattern regarding the impact of CTEPH on OSI (Fig. 8d). To further investigate temporal abnormalities in intralobar flow dynamics, we considered the combination of TAWSS and OSI (Fig. 9) represented by the dimensionless parameter ϕ. Tsubata et al. (2023) suggested that the combination of decreased WSS and increased OSI can contribute to thrombogenicity. Our results indicate that the percentage of vessels that had low TAWSS (< 5dyne/cm^2^) and high OSI (> 0.05) increased from baseline to CTEPH in all subjects. When the vascular endothelium experiences a decrease in wall shear stress and an increase in OSI, it inhibits the production and release of nitric oxide, a potent vasodilator (Terada et al. 2016) As a result, the abnormal flow dynamics as seen in our study may be linked to impaired vasodilation and vasoconstriction in the pulmonary arterioles.

### Limitations

Several limitations exist in our study, notably the small sample size, which contributed to heterogeneity. Limited image resolution in some subjects, especially one subject at baseline and a different subject after CTEPH, resulted in challenges extracting accurate subject-specific arterial networks and changes in those arterial networks with CTEPH. Since time-series pressure data were not recorded for either baseline or CTEPH conditions, we calibrated our model based on the scalar values of systolic and diastolic pressures (as well as scalar values of systolic and diastolic MPA area and time series LPA and RPA flow). The inclusion of time-series MPA pressure data would increase model fidelity. Finally, we assumed Blunt-like flow with a power law radial distribution for all arteries and held the power fixed at g=9. However, the range of Womersely number in the models was 2 to 40. As a consequence, in the small arteries, which will have lower Womersely numbers, TAWSS is overestimated. The impact on OSI is difficult to predict a priori; comparison to Tsubata et al. (2023) suggests OSI is underestimated in our model. Adapting g to local diameter or using an explicit Womersley profile in in the 1D CFD model would increase model complexity and solution time but increase accuracy.

## Conclusion

The impact of CTEPH on hemodynamic and vascular mechanical parameters that cannot be measured directly were examined using a subject-specific 1D CFD modeling framework. We calibrated the model to RHC- and MRI-derived measurements of pressure, area, and flow from a previously published large animal model of CTEPH. Our analysis revealed that CTEPH increased arterial stiffness and decreased TAWSS, and that the combination of decreased TAWSS and increased OSI in intralobar arteries may correlate with CTEPH progression. Our experimental-computational framework shows potential as a patient-specific simulator of both extralobar and intralobar pulmonary hemodynamics within the context of pulmonary hypertension. By leveraging advanced modeling techniques with model calibration, we can gain a more wholistic understanding of CTEPH, which could ultimately lead to more targeted interventions that address the specific needs of individuals with CTEPH.
